# Editorial: Brain and Somatization Symptoms in Psychiatric Disorders, Volume II

**DOI:** 10.3389/fpsyt.2022.881245

**Published:** 2022-04-08

**Authors:** Xiaoya Fu, Fengyu Zhang, Manli Huang, Lulu Zhang, Wenbin Guo

**Affiliations:** ^1^Department of Psychiatry, and National Clinical Research Center for Mental Disorders, The Second Xiangya Hospital of Central South University, Changsha, China; ^2^Global Clinical and Translational Research Institute, Bethesda, MD, United States; ^3^Beijing Huilongguan Hospital, Peking University Huilongguan Clinical Institute, Beijing, China; ^4^The Key Laboratory of Mental Disorder's Management, Department of Psychiatry, The First Affiliated Hospital, Zhejiang University School of Medicine, Hangzhou, China; ^5^Department of Psychiatry, Guangzhou First People's Hospital, The Second Affiliated Hospital of South China University of Technology, Guangzhou, China; ^6^Department of Psychiatry, The Third People's Hospital of Foshan, Foshan, China

**Keywords:** structural MRI, functional MRI, somatic symptoms, depression, anxiety

Neuroimaging techniques, such as magnetic resonance imaging (MRI), near-infrared spectroscopy, and single photon emission computed tomography (SPECT), are good approaches to identifying neural correlates associated with the onset and development of psychiatric disorders, which might be potential biomarkers for clinical diagnosis and measuring treatment response (1, 2).

Frequently, patients with psychiatric disorders coincide with somatic symptoms, especially in patients with depression and anxiety. About half of patients with major depressive disorder (MDD) reported multiple medical unexplained somatic symptoms (3). Half of patients attending the psychiatric outpatient's department had psychiatric disorders comorbid with at least one chronic somatic condition and 45% had more than two somatic conditions (4). The existence of somatic symptoms in psychiatric disorders often signals a more severe psychological state and a worse outcome, which markedly impairs the life quality of patients (5). Although quite common in psychiatric diseases, somatic symptoms are regarded as part of disease symptoms or side effects of therapy in most cases and rarely singled out to talk about their effects on neurocognitive tasks and brain function. Therefore, few studies focusing the neuropathology underlying somatic symptoms in psychiatric disorders and the effects of their interaction on neurocognitive performance and brain function.

This special issue is a continuum of our previous topic in order to help understand the neuropathy underlying somatic symptoms in psychiatric disorders (6). In this issue, a total of 4 papers that passed the preview process were published, including three research articles and one case report. One study focused on the effect of somatic symptoms on neurocognitive performance and the others were investigations on brain structure and function in neuropsychiatric abnormalities which are relatively uncommon.

Suzuki et al. used SPECT to study the pathophysiology of akathisia, a symptom of subjective restlessness and objective hyperactivity induced by psychotropic medications. They reported a case of 66-year-old woman with MDD, accompanied by chronic akathisia. Under the treatment of electroconvulsive therapy (ECT), her depressive symptoms alleviated, and the akathisia disappeared. SPECT was used before and after the ECT treatment to measure her regional cerebral blood flow.

Huang et al. investigated the effect of betel quid (BQ) on brain network dynamics. BQ is a mixture of substance widely consumed in Asia. As a psychoactive substance, its effect on dynamic brain network remains vague. This study found that BQ-dependent patients had severe depressive and anxiety symptoms, indicated by higher scores in Beck Depression Inventory and Beck Anxiety Inventory. After chewing BQ, both BQ-dependent subjects and participants in the control group showed higher global switching rate, indicating a more considerable temporal fluctuations of brain network organization.

Liao et al. showed their interest in congenital amusia (CA), a disorder with impairments in pitch perception and pitch memory. In this study, patients with CA performed worse in discriminating melodic and temporal dimensions of music and musical memory. This study used surface-based analyses and revealed the structural abnormalities of the right caudal middle frontal gyrus and right pars triangularis gyrus in patients with CA.

The study of Daming et al. investigated the influence of somatic symptoms on emotional memory in patients with major depressive disorder. This is the only study concerning the somatic problem in adolescence in this special issue. Patients with depression tend to remember negatively valenced stimuli or fail to demonstrate positive bias (7, 8). This study found lower valence rating and recognition accuracy for neutral and positive information depressed adolescences, suggesting that depressed adolescences failed to show protective bias toward neutral and positive contexts and had a general impairment of attention. The depressed adolescences with somatic symptoms were more sensitive to negative information than those without somatic symptoms and participants in the control group.

Generally, these studies have exhibited valuable explorations into brain and somatization symptoms in neuropsychiatric disorders. Despite prevalent in psychiatric disorders, somatic symptoms received relatively limited concerns. Neuroimaging techniques are powerful tools to examine brain structural and functional patterns in patients. We hope more clinicians and related researchers could pay more attention to somatic symptoms in psychiatric disorders and improve understandings of the neuropathological changes under the effect of somatic symptoms.

## Author Contributions

All authors listed have made a substantial, direct, and intellectual contribution to the work and approved it for publication.

## Funding

This study was supported by grants from the National Natural Science Foundation of China (grant no. 82171508), Natural Science Foundation of Hunan (grant no. 2020JJ4784), and Science and Technology Program of Hunan Province (grant no. 2020SK53413).

## Conflict of Interest

The authors declare that the research was conducted in the absence of any commercial or financial relationships that could be construed as a potential conflict of interest.

## Publisher's Note

All claims expressed in this article are solely those of the authors and do not necessarily represent those of their affiliated organizations, or those of the publisher, the editors and the reviewers. Any product that may be evaluated in this article, or claim that may be made by its manufacturer, is not guaranteed or endorsed by the publisher.
